# RCMF: a robust collaborative matrix factorization method to predict miRNA-disease associations

**DOI:** 10.1186/s12859-019-3260-0

**Published:** 2019-12-24

**Authors:** Zhen Cui, Jin-Xing Liu, Ying-Lian Gao, Chun-Hou Zheng, Juan Wang

**Affiliations:** 1 School of Information Science and Engineering, Qufu Normal University, Rizhao, 276826 China; 2 Co-Innovation Center for Information Supply & Assurance Technology, Anhui University, Hefei, 230601 China; 3 Qufu Normal University Library, Qufu Normal University, Rizhao, 276826 China

**Keywords:** MiRNA-disease association prediction, L_2,1_-norm, Collaborative regularization, Matrix factorization

## Abstract

**Background:**

Predicting miRNA-disease associations (MDAs) is time-consuming and expensive. It is imminent to improve the accuracy of prediction results. So it is crucial to develop a novel computing technology to predict new MDAs. Although some existing methods can effectively predict novel MDAs, there are still some shortcomings. Especially when the disease matrix is processed, its sparsity is an important factor affecting the final results.

**Results:**

A robust collaborative matrix factorization (RCMF) is proposed to predict novel MDAs. The L_2,1_-norm are introduced to our method to achieve the highest AUC value than other advanced methods.

**Conclusions:**

5-fold cross validation is used to evaluate our method, and simulation experiments are used to predict novel associations on Gold Standard Dataset. Finally, our prediction accuracy is better than other existing advanced methods. Therefore, our approach is effective and feasible in predicting novel MDAs.

## Background

A short class of non-coding RNAs called miRNAs, whose length is generally 19 to 25 nt. They usually regulate gene expression and protein production [1–7]. Since the first two miRNAs lin-4 and let-7 were discovered in 1993 and 2000, respectively [8, 9]. Thousands of miRNAs have been detected by biologists from nematodes to human eukaryotes [10, 11]. The latest miRNA database version miRBase contains 26,845 entries and more than 2000 human miRNAs are detected [12–14]. It is worth noting that with the development of bioinformatics, more researchers are starting to focus on the function of miRNAs. In addition, miRNAs begin to play an important role in biological processes such as proliferation, cell differentiation, viral infection, and signal transduction [15]. Moreover, some miRNAs are closely related to human diseases [16–18]. For example, mir-433 will upregulate the expression of GRB2 in gastric cancer, which is a known tumor-associated protein [19]. And in every pediatric brain tumor type, mir-25, mir-129, and mir-142 are differentially expressed [20]. Considering the strong association between miRNA and disease, all their potential associations should be explored [15, 21]. In medicine, the advantage is that it can promote the diagnosis and treatment of some complex diseases [22–25]. However, predicting MDAs is time-consuming and expensive. Only a few novel associations are discovered and used in clinical medicine each year, and most of the associations are not be discovered by researchers. Therefore, it is imminent to improve the accuracy of prediction results.

In previous studies, functionally similar miRNAs always appear in similar diseases [26, 27]. Based on such theory, more and more computational methods and models are proposed for identifying novel miRNA-disease associations (MDAs) [13]. However, these methods have some shortcomings more or less. For example, Jiang et al. proposed an improved disease-gene prediction model [28]. They introduced the principle and use of hypergeometric distribution. And then they analyzed the actual effect in the prediction model. Moreover, different datasets are used to predict novel MDAs, including the known human miRNA-disease data, miRNA functional similarity data and disease semantic similarity data. However, the shortcoming of this model is the excessive dependence on neighbor miRNA data. Chen et al. proposed a method HGIMDA (Heterogeneous Graph Inference miRNA-Disease Association) to predict novel MDAs [29]. It is worth noting the known miRNA-disease associations, miRNA functional similarity, disease semantic similarity, and Gaussian interaction profile kernel similarity for diseases and miRNAs are integrated into this method. The benefit is that the accuracy of the algorithm is improved to some extent. The functional relationship between miRNA targets and disease genes in PPI (Protein–Protein Interaction) networks are considered by researchers. Shi et al. proposed a computational method to predict MDAs by performing random walk [30]. They used PPIs, the miRNA-target interactions and disease-gene associations to identify potential MDAs. However, the model strongly depended on the miRNA-target interactions with high rate of false-positive and high false-negative results [31]. Considering this disadvantage, Chen et al. proposed the RWRMDA (Random Walk with Restart for MiRNA-disease association) model [32]. Their approach was to map all miRNAs to a miRNA functional similarity network. Then, random walk with restart method was implemented until they got stable probability [33]. Finally, all candidate miRNAs will be sorted according to the probability of stability. Moreover, the method was the first global network-based method. Xuan et al. proposed a HDMP method [34]. The mothed was based on weighted k-nearest-neighbors. The phenotype similarity and semantic similarity between diseases were used to calculate the miRNAs functional similarity matrix. However, the simple ranking of k-nearest-neighbors was not always reliable for prediction. So Chen et al. proposed a new method of ranking-based KNN called RKNNMDA to identify potential MDAs [34]. These previously similarity-based sorted neighbors were re-ranked to get better prediction results. Recently, matrix factorization methods have been used to identify novel MDAs. The advantage is that these methods can better handle missing associations. Shen et al. proposed a matrix factorization model based collaborative matrix factorization to predict novel MDAs [10]. Matrix factorization method takes one input matrix and tries to obtain two other matrices, then the two matrices are multiplied to approximate the input matrix. Gao et al. proposed a dual-network sparse graph regularized matrix factorization method (DNSGRMF) to predict novel MDAs and obtained better experimental results [35]. However, this method does not necessarily solve the overfitting problem very well. Chen et al. developed a computational model of ELLPMDA (Ensemble Learning and Link Prediction for miRNA-Disease Association) to predict novel MDAs [36]. The miRNA-disease association, miRNA functional similarity, disease semantic similarity and Gaussian profile kernel similarity for miRNAs and diseases were integrated, they used the integrated similarity network and utilized ensemble learning. Three classical algorithms based on similarity are combined to obtain better prediction results. However, even such an excellent method still has some shortcomings, such as excessive ensemble learning will bring more noise. Gao et al. proposed a Nearest Profile-based Collaborative Matrix Factorization (NPCMF) method to predict potential miRNA-disease associations [37]. More importantly, this method has achieved the highest prediction accuracy so far.

In this paper, a simple yet effective matrix factorization model is proposed. Its main function is to predict new MDAs based on existing MDAs. Considering that the missing associations will have a negative impact on the predictions, a pre-processing step is used to solve this problem. The main purpose of this pre-processing method is to try to weight K nearest known neighbors (WKNKN) [38, 39]. It is worth noting that the L_2,1_-norm is introduced in the collaborative matrix factorization (CMF) method. And the L_2,1_-norm can avoid over-fitting and eliminate some unattached disease pairs [40, 41]. We also use Gaussian interaction profile kernel similarity to get the network similarity of miRNAs and the network similarity of diseases. Therefore, the final prediction accuracy is greatly improved. Meanwhile, 5-fold cross validation is used to evaluate our experimental results. Our proposed method is superior to other methods. In addition, a simulation experiment is conducted to predict novel associations.

## Materials

### MDAs dataset

The information about associations between miRNA and disease is obtained from HMDD [42], including 383 diseases, 495 miRNAs and 5430 experimentally confirmed human miRNA-diseases associations. And it is a Gold Standard Dataset. The dataset contains three matrices: **Y** ∈ *ℝ*^*n* × *m*^, **S**_m_ ∈ *ℝ*^*n* × *n*^ and **S**_*d*_ ∈ *ℝ*^*m* × *m*^. In addition, **Y** is an adjacency matrix. In the adjacency matrix, there are *n* miRNAs as rows and *m* diseases as columns. If miRNA *D*(*i*) is associated with disease *d*(*j*), the entity **Y**(*D*(*i*), *d*(*j*)) is 1, otherwise 0. The matrix **Y** is used as the original input matrix. **Y** is decomposed into two latent feature matrices, and the product of the two latent feature matrices is used to approximate **Y**. Table 1 lists the specific information for the dataset.

**Table 1:** MiRNAs, Diseases, and Associations in Gold Standard Dataset

Datasets	MiRNAs	Diseases	Associations
Gold Standard Dataset	495	383	5430

### MiRNA functional similarity

Considering the assumption that similarly functioning miRNAs have similar diseases, Wang *et.al*. proposed a method for calculating the similarity scores of RNA functions [26]. And the miRNA functional similarity scores are downloaded from http:// www.cuilab.cn/files/images/cuilab/misim.zip. The matrix **S**_m_ is represented miRNA function similarity network. The functional similarity score between miRNA *m*(*i*) and *m*(*j*) can be represented **S**_m_(*m*(*i*), *m*(*j*)). The **S**_m_ matrix is also used as an input matrix, which represents the functional similarity of miRNA pairs. Among them, each miRNA has a similarity score of 1 to itself.

### Disease semantic similarity

In this work, Directed Acyclic Graph (DAG) is used to describe the diseases. DAG(*DD*) = (*d*, *T*(*DD*), *E*(*DD*)) is used to describe disease *DD*, where *T*(*DD*) is the node set and *E*(*DD*) is the corresponding links set [26]. **S**_d_ is represented disease semantic similarity network. The semantic value of disease *DD* in DAG(*DD*) formula is defined as:

1 $$ DV1(DD)=\sum \limits_{d\in T(DD)}D{1}_{DD}(d), $$

2 $$ D{1}_{D\mathrm{D}}(d)=\Big\{{\displaystyle \begin{array}{l}1\kern15.30002em if\kern0.3em d= DD\kern0.1em \\ {}\max \left\{\varDelta \ast D{1}_{DD}\left({d}^{\hbox{'}}\right)|{d}^{\hbox{'}}\in childrenof\kern0.1em d\right\}\kern0.5em if\kern0.3em d\ne DD,\end{array}} $$

where *Δ* is represented the semantic contribution factor. Generally, the semantic contribution of disease *DD* to itself is 1. Based on previous research [43], we set *Δ* to 0.5. It is worth noting that the further the distance between *DD* and other disease, the smaller the semantic contribution score. Therefore, disease terms contribute the same score to the semantic value of the disease *DD* in the same layer. Finally, if the two diseases *d*(*i*) and *d*(*j*) have a larger common part of the DAGs, then the two diseases have a greater similarity score. The disease semantic similarity can be defined as follows:

3 $$ {\mathbf{S}}_{\mathrm{d}}\left(d(i),d(j)\right)=\frac{\sum_{t\in T\left(d(i)\right)\cap T\left(d(j)\right)}\left(D{1}_{d(i)}(t)+D{1}_{d(j)}(t)\right)}{DV1\left(d(i)\right)+ DV1\left(d(j)\right)}, $$

where **S**_d_ is the disease semantic similarity matrix. In addition, the **S**_d_ matrix is also used as an input matrix with **Y** and **S**_m_. Similar to the **S**_m_ matrix, each disease has its own semantic similarity score of 1. Therefore, the two feature matrices decomposed by **Y** are controlled by the **S**_m_ matrix and the **S**_d_ matrix.

## Methodology

### Problem formalization

Formally, the known associations **Y**(*m*(*i*), *d*(*j*)) of miRNA *m*(*i*) associated with disease *d*(*j*) are considered to be a matrix factorization model. First, the input associations matrix **Y** is decomposed into two low rank latent feature matrices **A** (for miRNAs) and **B** (for diseases). Then, some constraints are added to the two low rank matrices [44]. Specifically, the L_2,1_-norm is added to the latent feature matrix **B** (for diseases). Finally, the specific matrices of **A** and **B** are obtained by using some update rules. It is worth noting that we need a prediction matrix that is derived from the product of **A** and **B**. Considering the stronger association of miRNAs with diseases, the correlation score between them is higher. So, the miRNA-disease pairs **Y**(*m*(*i*), *d*(*j*)) are ranked from high to low.

### Robust collaborative matrix factorization (RCMF)

The traditional CMF is an effective method for predicting novel MDAs [10]. Collaborative filtering is used by CMF. The objective function of CMF is given as follows:

4 $$ {\displaystyle \begin{array}{l}{\min}_{\mathbf{A},\mathbf{B}}={\left\Vert \mathbf{Y}-\mathbf{A}{\mathbf{B}}^T\right\Vert}_F^2+{\lambda}_l\left({\left\Vert \mathbf{A}\right\Vert}_F^2+{\left\Vert \mathbf{B}\right\Vert}_F^2\right)\\ {}+{\lambda}_d{\left\Vert {\mathbf{S}}_m-\mathbf{A}{\mathbf{A}}^{\mathrm{T}}\right\Vert}_F^2+{\lambda}_t{\left\Vert {\mathbf{S}}_{\mathrm{d}}-\mathbf{B}{\mathbf{B}}^T\right\Vert}_F^2,\end{array}} $$

where ‖⋅‖_*F*_ is Frobenius norm, *λ*_*l*_, *λ*_*d*_ and *λ*_*t*_ are non-negative parameters.

However, although the **B** matrix is a low rank matrix, it is not sparse enough. In fact, **B** is indeed sparse. But we want to get the **B** matrix better, we use the L_2,1_-norm to constrain the latent feature matrix **B** of the disease. Because the L_2,1_-norm can achieve row sparse, the L_2,1_-norm can better remove the meaningless elements of the **B** matrix. For matrix **B**, overfitting problems may be generated to reduce the accuracy of the prediction in predicting novel MDAs.

Therefore, to overcome this problem, a robust collaborative matrix factorization method named RCMF is proposed to predict MDAs. The L_2,1_-norm is introduced to the RCMF method to solve over-fitting problems [45, 46]. In this paper, the dataset used in the experiment, the number of diseases is less than the number of miRNAs, we are more concerned about which miRNAs are likely to be associated with the diseases. Therefore, we apply the L_2,1_-norm on the potential feature matrix **B** of the disease to make the **B** matrix sparse. The advantage is that more miRNAs can be accurately matched to the disease to improve the accuracy of prediction. The interaction matrix **Y** is decomposed into two matrices **A** and **B**, where **AB**^*T*^ ≈ **Y**. RCMF uses two collaborative regularization terms to constrain **A** and **B**. Specifically, these two regularization terms require similar miRNAs or diseases potential feature vectors to be similar, and dissimilar miRNAs or diseases potential feature vectors are not similar, respectively [38]. Where **S**_m_ ≈ **AA**^*T*^ and **S**_d_ ≈ **BB**^*T*^. Therefore, the objective function of RCMF can be written as:

5 $$ {\displaystyle \begin{array}{l}{\min}_{\mathbf{A},\mathbf{B}}={\left\Vert \mathbf{Y}-\mathbf{A}{\mathbf{B}}^{\mathrm{T}}\right\Vert}_F^2+{\lambda}_l\left({\left\Vert \mathbf{A}\right\Vert}_F^2+{\left\Vert \mathbf{B}\right\Vert}_F^2\right)+{\lambda}_l{\left\Vert \mathbf{B}\right\Vert}_{2,1}\\ {}+{\lambda}_d{\left\Vert {\mathbf{S}}_{\mathrm{m}}-\mathbf{A}{\mathbf{A}}^{\mathrm{T}}\right\Vert}_F^2+{\lambda}_t{\left\Vert {\mathbf{S}}_{\mathrm{d}}-\mathbf{B}{\mathbf{B}}^{\mathrm{T}}\right\Vert}_F^2,\end{array}} $$

where ‖⋅‖_*F*_ is Frobenius norm, ‖⋅‖_2, 1_ is L_2,1_-norm, *λ*_*l*_, *λ*_*d*_ and *λ*_*t*_ are non-negative parameters. Based on previous research [38], the grid search method is used to perform the selection of optimal parameters, where *λ*_*l*_ ∈ {2^−2^, 2^−1^, 2^0^, 2^1^} and *λ*_*d*_/*λ*_*d*_ ∈ {0, 10^−4^, 10^−3^, 10^−2^, 10^−1^}. In order to find the latent feature matrices **A** and **B**, an approximate model of the matrix **Y** is constructed in the first term. In the second term, the Tikhonov regularization can minimize the norms of **A**, **B**. The L_2,1_-norm is applied on **B** in the third term. And this advantage is able to increase the sparsity of the disease matrix and eliminate undesired disease pairs. The last two regularization terms represent the minimization of squared error between **S**_m_ (**S**_d_) and **AA**^*T*^ (**BB**^*T*^).

#### Initialization of **A** and **B**

**A** and **B** are initialized to use the SVD (Singular Value Decomposition) method for the input MDAs matrix **Y**. The initialization formula can be written as:

6 $$ \left[\mathbf{U},\mathbf{S},\mathbf{V}\right]=\mathrm{SVD}\left(\mathbf{Y},k\right),\mathbf{A}=\mathbf{U}{\mathbf{S}}_k^{1/2},\mathbf{B}=\mathbf{V}{\mathbf{S}}_k^{1/2}, $$

where **S**_*k*_ is a diagonal matrix, which contains the *k* largest singular values.

#### Optimization algorithm

**Fig. 1:**
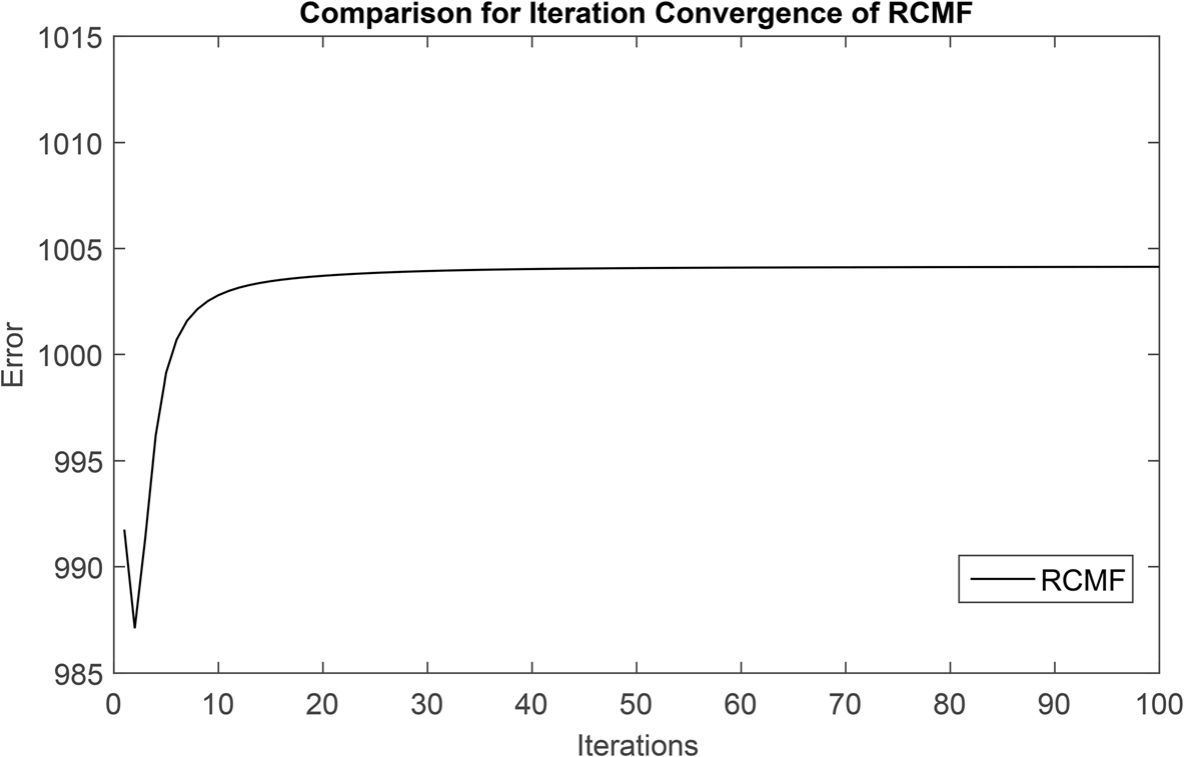
Convergence analysis of RCMF method

**A** and **B** are updated using least squares in this study. First, *F* is represented as the objection function of RCMF method. Then, *∂F*/*∂***A** and *∂F*/*∂***B** are set to be 0, respectively. **A** and **B** are continued to use the least squares until convergence. Figure 1 shows the convergence of the RCMF method. We perform the RCMF method on the dataset used in the experiment, where the x-axis represents the number of iterations and the y-axis represents the error. As can be seen from Fig. 1, after 50 iterations, the curve begins to converge on a straight line, which proves that our method begins to converge after 50 iterations. In addition, *λ*_*l*_, *λ*_*d*_ and *λ*_*t*_ are automatically determined. The optimal parameter values are obtained when cross validating the training set. The update rules of **A** and **B** can be written as:

7 $$ \mathbf{A}=\left(\mathbf{YB}+{\lambda}_d{\mathbf{S}}_{\mathrm{m}}\mathbf{A}\right){\left({\mathbf{B}}^{\mathrm{T}}\mathbf{B}+{\lambda}_l{\mathbf{I}}_{\mathrm{k}}+{\lambda}_d\mathbf{A}{\mathbf{A}}^{\mathrm{T}}\right)}^{-1}, $$

8 $$ \mathbf{B}=\left({\mathbf{Y}}^{\mathrm{T}}\mathbf{A}+{\lambda}_t{\mathbf{S}}_{\mathrm{d}}\mathbf{B}\right){\left({\mathbf{A}}^{\mathrm{T}}\mathbf{A}+{\lambda}_l{\mathbf{I}}_{\mathrm{k}}+{\lambda}_t{\mathbf{B}}^{\mathrm{T}}\mathbf{B}+{\lambda}_l\mathbf{D}{\mathbf{I}}_{\mathrm{k}}\right)}^{-1}, $$

where **D** is a diagonal matrix with the *i*-th diagonal element as *d*_*ii*_ = 1/2‖(**B**)^*i*^‖_2_. Based on these update rules, we first calculate the maximum time complexity required to perform the iterative steps, and then we conclude that the final time complexity of RCMF method is *O*(*nmk*), where *n* is the number of miRNAs, *m* is the number of diseases and *k* is the number of singular values in the SVD. Therefore, the algorithm of RCMF is as follows:

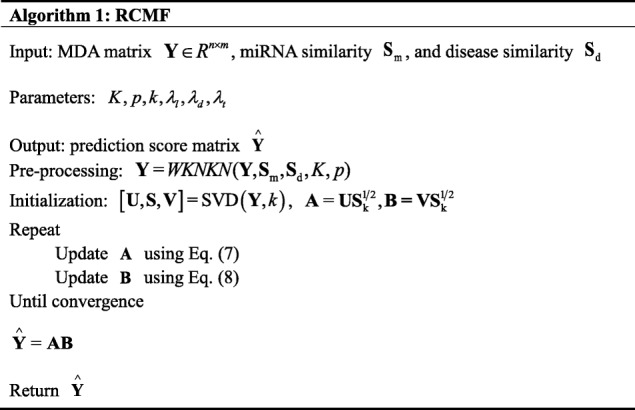



## Results

### Cross validation experiments

In this study, our experiments are compared to the previous advanced methods CMF [10], HDMP [33], WBSMDA [47], MKRMDA [31], HAMDA [48] and ELLPMDA [36]. For each method, 5-fold cross validation is conducted 100 times. However, the WKNKN pre-processing steps is performed before running our method. This can solve the problem of missing unknown values. At the same time, it can also improve the accuracy of prediction to some extent.

In general, AUC (Area Under the Curve) is used as a reasonable indicator when evaluating the predictive performance of a method. The popular indicator of AUC is also used to evaluate our approach in this study. The area under the ROC (Receiver Operating Characteristic) curve is considered to be AUC. In other words, the value of this area will not be greater than 1. AUC values between 0.5 and 1 are normal and reasonable. Once below 0.5, the method will have no meaning at all. Before running cross validation, the miRNA-disease pairs are randomly removed in the input MDAs matrix **Y**. Doing this is a comprehensive assessment of our approach by increasing the difficulty of prediction [49]. This way is called CV-p (Cross Validation pairs).

#### Association prediction under CV-p

Table 2 lists the experimental results at CV-p. The AUC average of 100 times 5-fold cross validation is used as the final AUC score. It is worth noting that AUC is known to be insensitive to skewed class distributions [50]. The gold standard miRNA disease dataset is highly unbalanced in this study. One problem is that there are more negative factors than positive ones. Thus, AUC is a more suitable measure for other methods. As shown in Table 2, the AUC values for each method are counted, including the highest AUC in bold, and standard deviations are given in (parentheses).

**Table 2:** AUC Results of cross validation experiments

Methods	Gold Standard Dataset
WBSMDA	0.8185(0.0009)
HDMP	0.8342(0.0010)
CMF	0.8697(0.0011)
MKRMDA	0.8894(0.0015)
HAMDA	0.8965 (0.0012)
ELLPMDA	0.9193(0.0002)
RCMF	0.9345(0.0004)

**Fig. 2:**
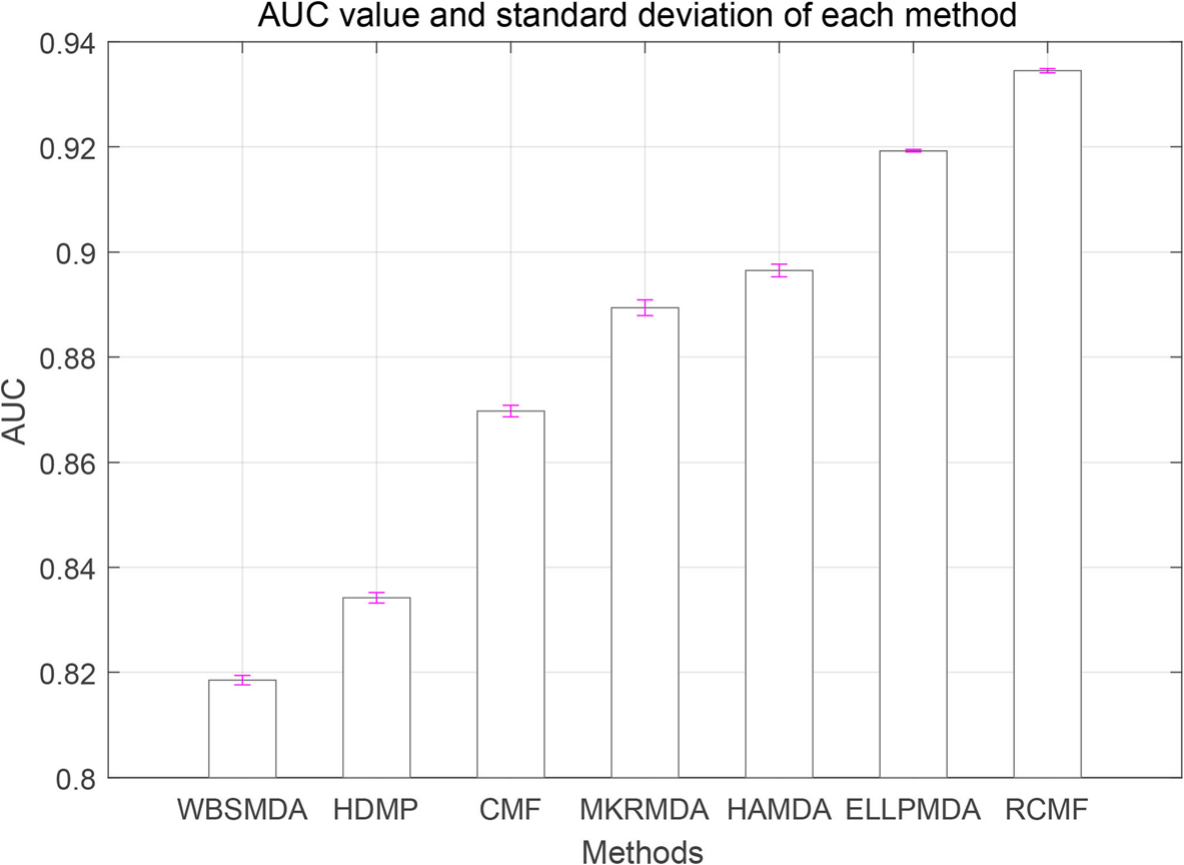
AUC value on Gold Standard Dataset

As listed in Table 2, our proposed method RCMF achieves an AUC of 0.9345 on Gold Standard Dataset, which is 1.52% higher than ELLPMDA with an AUC of 0.9193. The AUC value of the WBSMDA method is the lowest, and our method is 11.6% higher than it. Also, our method is 6.48% higher than the traditional CMF method. Therefore, our proposed is better than other existing methods. Figure 2 visually shows the AUC level of each method.

### Comprehensive prediction for novel MDAs

A simulation experiment is conducted in this subsection. Two cases are tested by our method, one is Esophageal Neoplasms, the other is Liver Neoplasms. Esophageal Neoplasms is very common in many areas of China, especially in northern China [51].. More information about the disease are published in http://www.omim.org/entry/133239. For Esophageal Neoplasms, the 30 miRNAs associated with it are removed. Then, the simulation is conducted to get the final prediction score matrix. Based on the predicted scores for this disease, the miRNAs associated with this disease are ranked from high to low. At the same time, whether the removed miRNA is successfully predicted and the novel associations are also needs to be counted. Twenty known associations are successfully predicted and five novel associations are predicted. Among the unknown associations, the three of five unknown associations are confirmed by the dbDEMC [52]. It is worth noting that hsa-mir-215 has the highest correlation with Esophageal Neoplasms. About hsa-mir-215, Fassan et al. have discovered this miRNA in 2011 related to Esophageal Neoplasms. They performed qRT-PCR and ISH analyses on two independent series of endoscopic biopsies (qRT-PCR) and esophagectomy specimens (ISH) [53]. In particular, hsa-mir-215 is significantly overexpressed during the pathogenesis of Esophageal Neoplasms. About hsa-mir-184, Kojima et al. discovered this miRNA in 2015 related to Esophageal Neoplasms. They conducted miRNA expression analysis by microarray [54]. By comparing Esophageal Neoplasms with normal samples, hsa-mir-184 is under-expressed in diseased samples. Table 3 lists the experimental results. And the known associations are in bold.

**Table 3:** Predicted MiRNAs for Esophageal Neoplasms

Rank	miRNA	Evidence	Rank	miRNA	Evidence
1	hsa-let-7a	known	16	hsa-mir-145	known
2	hsa-mir-100	known	17	hsa-mir-146a	known
3	hsa-mir-130a	known	18	hsa-mir-148a	known
4	hsa-let-7c	known	19	hsa-mir-617	known
5	hsa-mir-192	known	20	hsa-mir-758	known
6	hsa-mir-19a	known	21	hsa-mir-342	known
7	hsa-mir-21	known	22	hsa-mir-34a	known
8	hsa-mir-150	known	23	hsa-mir-34b	known
9	hsa-mir-205	known	24	hsa-mir-296	known
10	hsa-mir-22	known	25	hsa-mir-29c	known
11	hsa-mir-223	known	26	hsa-mir-215	dbDEMC
12	hsa-mir-25	known	27	hsa-mir-421	dbDEMC
13	hsa-mir-26a	known	28	hsa-mir-184	dbDEMC
14	hsa-mir-27a	known	29	hsa-mir-519a	Unconfirmed
15	hsa-mir-28	known	30	hsa-mir-610	Unconfirmed

Another case is Liver Neoplasms. It is the fifth most common cancer and the third most common cause of death from cancer worldwide [55]. More information about the disease are published in http://www.omim.org/entry/114550. For Liver Neoplasms, fifteen miRNAs associated with it are removed from the dataset while running our method. Then based on the predicted scores for this disease, the miRNAs associated with this disease are ranked from high to low. Finally, twelve known associations are successfully predicted. At the same time, three novel associations are predicted. And, all three are confirmed by dbDEMC. About hsa-mir-200b, hsa-mir-15b and hsa-mir-183, Naoki et al. have discovered this miRNA in 2012 related to Liver Neoplasms. In particular, hsa-mir-200b, hsa-mir-15b,and hsa-mir-183 are significantly overexpressed during the pathogenesis of Liver Neoplasms [56]. Table 4 lists the experimental results.

**Table 4:** Predicted MiRNAs for Liver Neoplasms

Rank	miRNA	Evidence
1	hsa-mir-372	known
2	hsa-mir-486	known
3	hsa-mir-10b	known
4	hsa-mir-122	known
5	hsa-mir-133b	known
6	hsa-mir-200a	known
7	hsa-mir-148b	known
8	hsa-mir-21	known
9	hsa-let-7b	known
10	hsa-mir-629	known
11	hsa-mir-24	known
12	hsa-mir-34c	known
13	hsa-mir-200b	dbDEMC
14	hsa-mir-15b	dbDEMC
15	hsa-mir-183	dbDEMC

According to the above simulation results, most known miRNAs are predicted. At the same time, some unknown miRNAs are also confirmed by dbDEMC. Therefore, our method can be used to predict novel MDAs and achieve excellent predictions.

## Discussion

### Sensitivity analysis from WKNKN

**Fig. 3:**
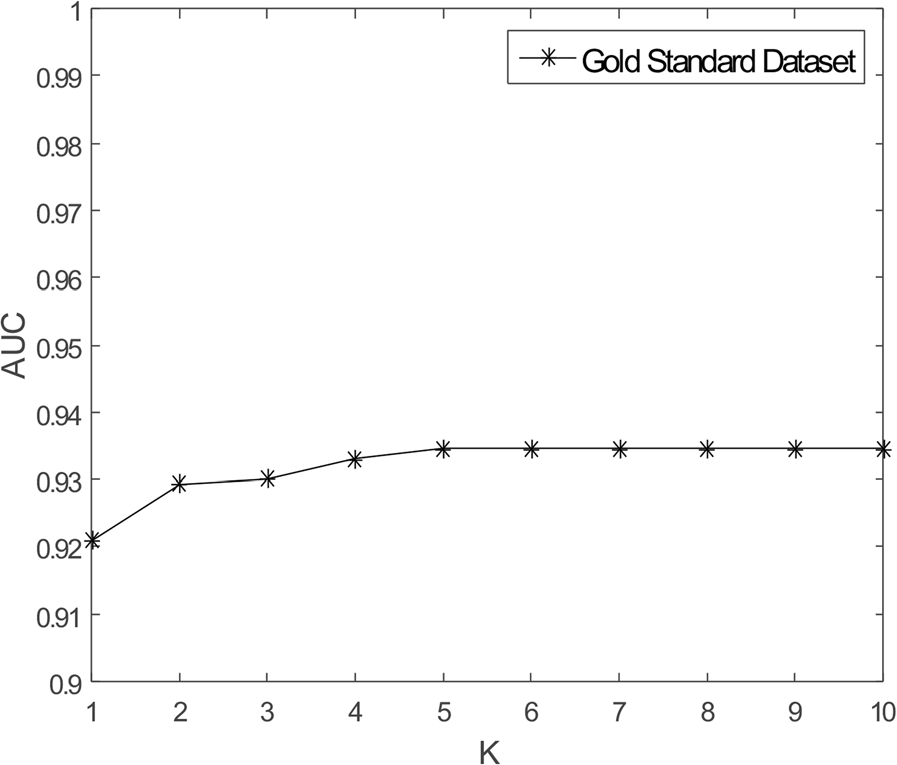
Sensitivity analysis for *K* under CV-p

**Fig. 4:**
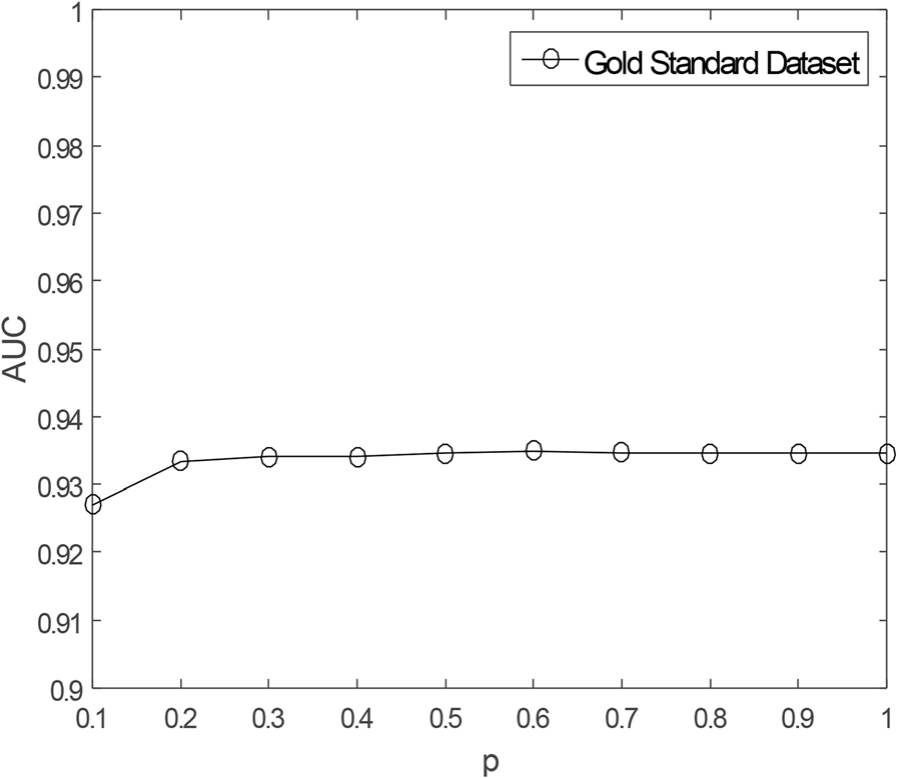
Sensitivity analysis for *p* under CV-p

**Fig. 5:**
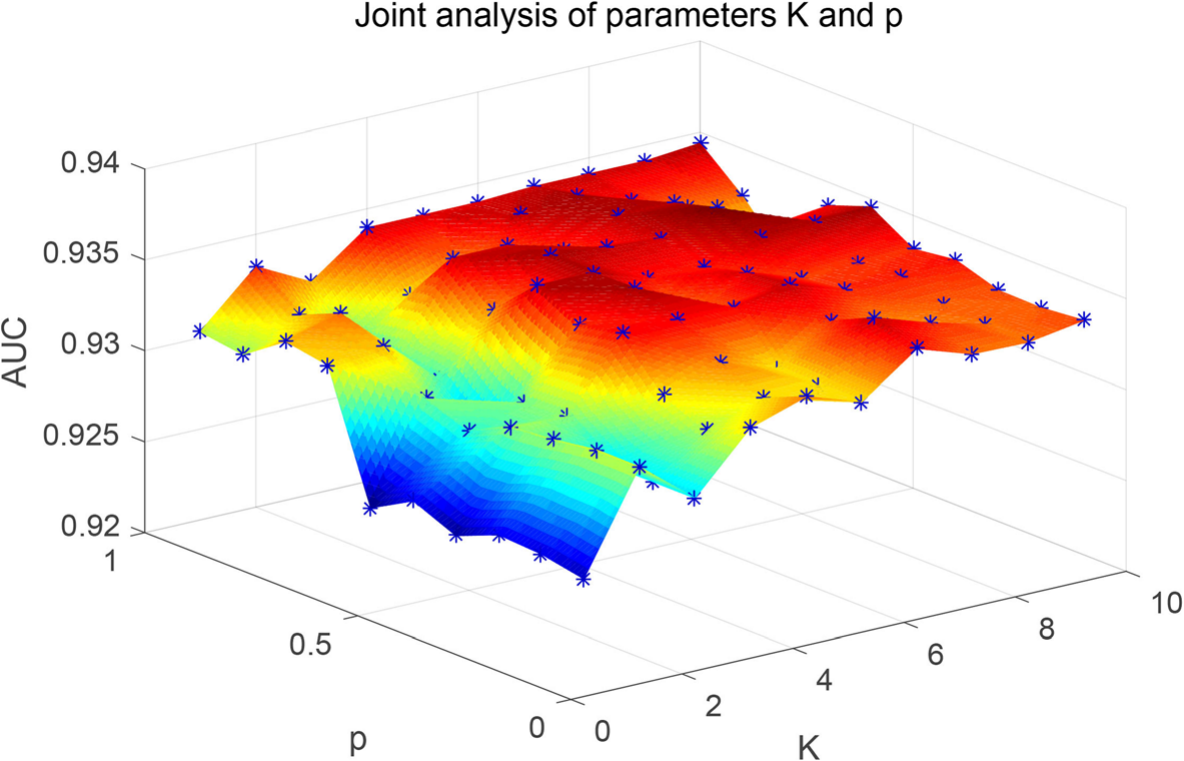
Joint sensitivity analysis of parameters *K* and *p*

As mentioned earlier in this study, there are some missing unknown associations in the matrix **Y**, so WKNKN method is used to minimize the error. *K* represents the number of nearest known neighbors and *p* represents a decay term where *p* ≤ 1. Before running RCMF method, the parameters *K* and *p* will be fixed. The sensitivity analysis of these two parameters is given by Figs. 3 and 4, respectively. It can be clearly seen from the figures that when *K* = 5, *p* = 0.7, the AUC tends to be stable. Furthermore, to more fully verify the sensitivity of these two parameters to AUC, their joint sensitivity analysis is shown in Fig. 5.

### Robust analysis of our method

**Fig. 6:**
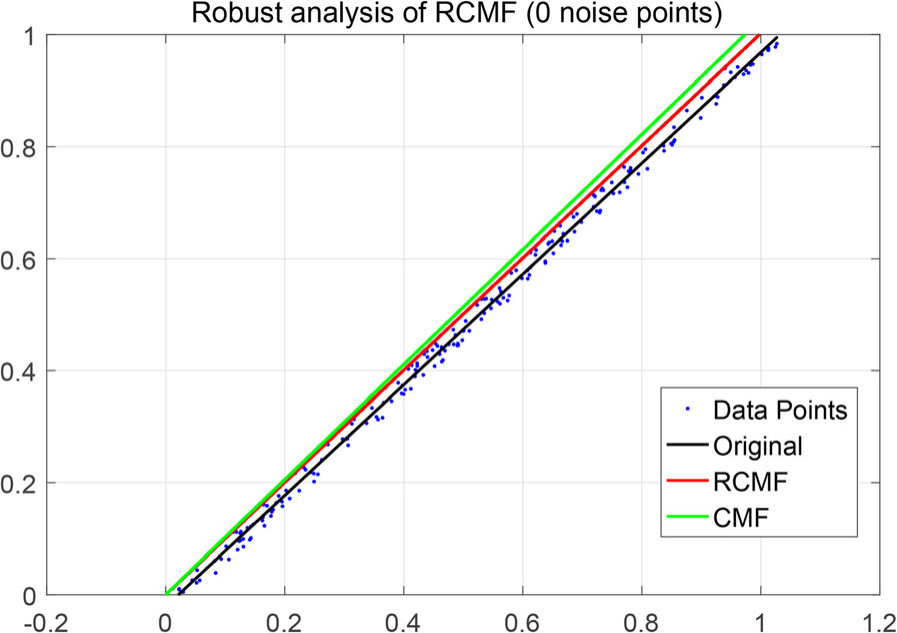
Robustness comparison between RCMF and CMF when there are 0 noise points

**Fig. 7:**
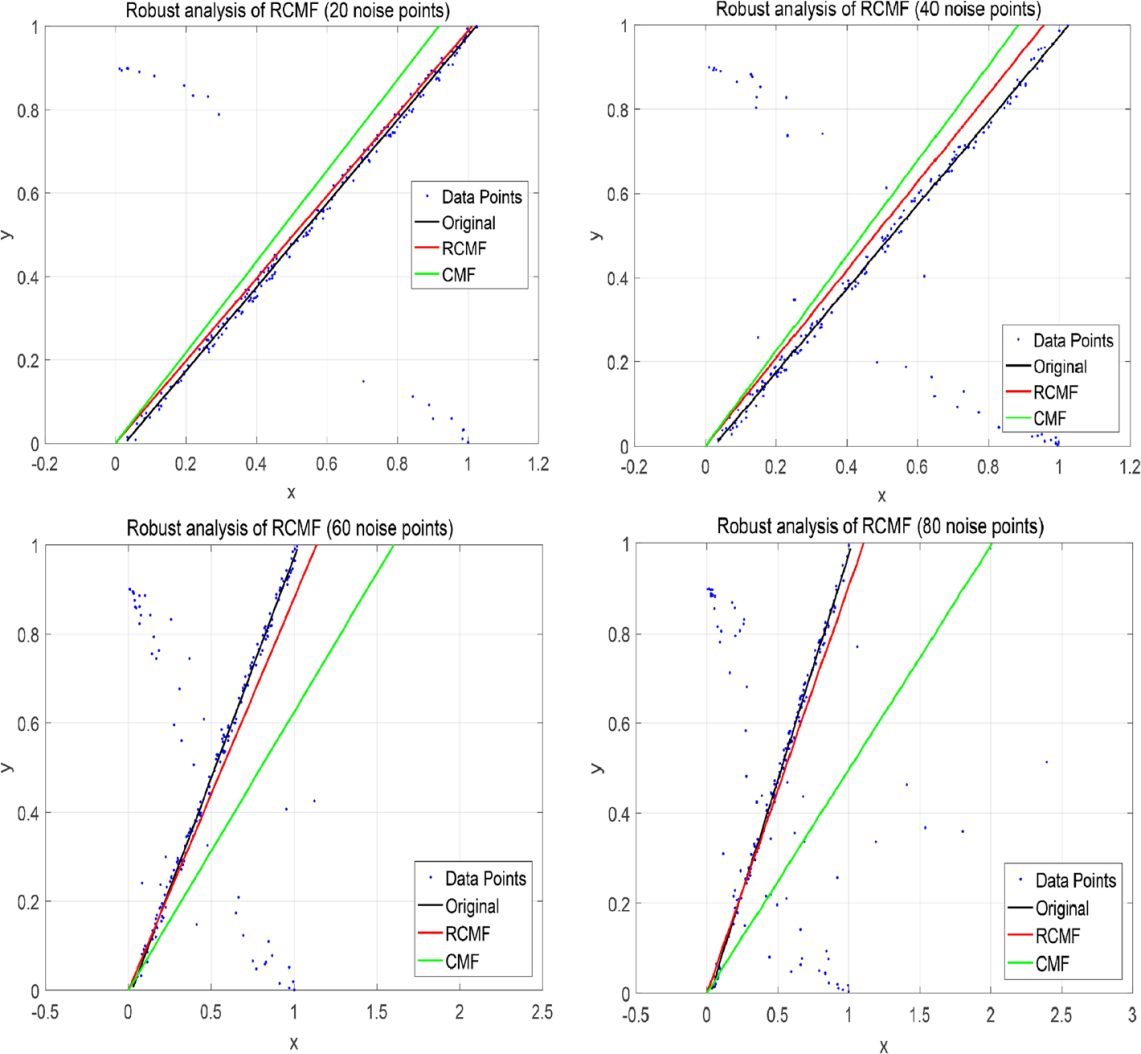
Robustness comparison between RCMF and CMF when there are 20, 40, 60 and 80 noise points, respectively

The L_2,1_-norm can increase the robustness of the algorithm. This is mainly reflected in the distinction between outliers in the dataset. In this section, we use a simulation dataset of 200 data points to verify the robustness of the algorithm. To illustrate RCMF’s ability to learn a subspace, we apply RCMF on a synthetic dataset composed of 200 two-dimensional data points. It is worth noting that all data points are distributed in a one-dimensional subspace, i.e., a straight line (*y* = *x*). In addition, both RCMF and CMF are applied to the synthetic data set for comparison. Specifically, we add different numbers of noise points to the simulation dataset to compare RCMF and CMF. Figures 6 and 7 show the data distribution of 0 noise points, 20 noise points, 40 noise points, 60 noise points and 80 noise points, respectively. As can be seen from Fig. 6, both RCMF and CMF remain stable when there are no noise points in the dataset. It can be seen from Fig. 7 that as the noise point increases, the CMF cannot continue to maintain stability but gradually shifts. It is worth noting that RCMF can still maintain the same state as the original data point due to the L_2,1_-norm. Even if the number of noise points is constantly increasing, RCMF is still unaffected by outliers. This proves that RCMF is robust.

## Conclusions

Abnormal expression of miRNA has a crucial impact in the development of complex human diseases. More and more diseases are confirmed by biologists to have a close relationship with miRNAs. In this paper, a novel computational model is proposed to predict MDAs. The most valuable contribution is that the L_2,1_-norm is added to the CMF. AUC value is used as a reliable indicator to evaluate our approach. Meanwhile, the excellent results are generated by our method.

More importantly, WKNKN is used as a pre-processing method. This step plays a crucial role in predicting MDAs. The best predictions are achieved by dealing with missing unknown associations.

In the future, more and more novel MDAs will be predicted and more datasets will be available. At the same time, more valuable MDA information will be published in public databases. In fact, there are many other methods to predict MDAs. RCMF is hoped to be helpful for MDA prediction and relevant miRNA research from the computational biology. In future work, we will continue to study more effective methods to predict novel MDAs.

## Data Availability

The datasets that support the findings of this study are available in https://github.com/cuizhensdws/L21-GRMF.

## Abbreviations

AUC: Area under the curve
CMF: Collaborative matrix factorization method
CV: Cross-validation
CV-p: Cross validation pairs
MDAs: MiRNA-disease associations
RCMF: Robust collaborative matrix factorization method
SVD: Singular value decomposition
